# Impact of resveratrol supplementation on clinical parameters and inflammatory markers in patients with chronic periodontitis: a randomized clinical trail

**DOI:** 10.1186/s12903-023-02877-4

**Published:** 2023-03-27

**Authors:** Shabnam Nikniaz, Farzane Vaziri, Reza Mansouri

**Affiliations:** 1 Department of Periodontics, School of Dentistry, Shahid Sadoughi University of Medical Sciences, Yazd, Iran; 2 Department of Immunology, faculty of medicine, Shahid Sadoughi University of Medical Sciences, Yazd, Iran

**Keywords:** Chronic periodontitis, Polyphenol, Dietary supplementation, Resveratrol

## Abstract

**Background:**

Periodontitis is one of the most common chronic inflammatory diseases in the world, which affects oral health. Resveratrol is a polyphenol with therapeutic effects on the inflammation caused by periodontal pathogens. This study aimed to evaluate the impact of resveratrol supplementation on clinical parameters and inflammatory markers in patients with chronic periodontitis.

**Methods:**

In this randomized, double-blind study, 40 chronic periodontitis patients underwent non-surgical therapy and were randomly assigned to two intervention and control groups, receiving either resveratrol supplements or a placebo for four weeks. Salivary levels of interleukin-8 (IL-8), interleukin-1β (IL-1β), and clinical parameters, including pocket depth (PD), clinical attachment level (CAL), plaque index (PI), and bleeding index (BI), were measured before and after the intervention.

**Results:**

The results showed that in both the case and control groups, after four weeks of using resveratrol, only plaque index (PI) was significantly different compared to the control group (P = 0.0001). However, there were no significant differences in the mean pocket depth (PD), clinical attachment loss (CAL), bleeding index (BI), and salivary levels of IL-8 and IL-1β between the two groups after the intervention.

**Conclusion:**

Resveratrol complement was helpful as an anti-inflammatory food supplement, along with other non-surgical periodontal treatments in chronic periodontitis patients.

**Supplementary Information:**

The online version contains supplementary material available at 10.1186/s12903-023-02877-4.

## Introduction

Periodontitis is an inflammatory disease that leads to the destruction of dental supporting tissues and tooth loss [1]. This immune inflammatory disorder originate from the formation of complex subgingival microbial biofilms and periodontal microbiota dysbiosis. One of the main pathogens in the initiation of periodontitis, Porphyromonas gingivalis, stimulates proinflammatory cytokines such as IL-1β, IL-8, IL-6, and TNFα, which penetrate the gingival connective tissue, cause a local inflammatory response [2–4], and increase the number and activity of polymorphonucleocytes (PMNs), in association with the production of cytokines. In addition, these PMNs produce reactive oxygen species (ROS) and trigger a defense response to infection [5]. Also, ROS can activate macrophages for the synthesis and secretion of inflammatory cytokines and hydrolytic enzymes; it also plays a crucial role in the destruction of periodontal tissues by inflammatory and catabolic activities [6].

Given the complex processes created by different pro-inflammatory and anti-inflammatory mediators, studies have investigated different strategies for regulating immune-inflammatory responses to periodontal diseases. Previous studies have shown that both non-steroid anti-inflammatory drugs (NSAIDs) and selective COX-2 inhibitors regulate the host immune-inflammatory activities [7–10]. However, systemic use of these drugs causes severe complications, decreasing patients’ compliance to use them [11].

Antibiotics have been widely used for treating periodontal diseases; however, using them frequently contributes to antibiotic resistance [11]. Recently natural components derived from plants, such as polyphenols and essential oil have been received attention for the treatment of periodontitis [12–15]. These natural compound have been interestingly evaluated to rejuvenate the oral cavity [16]. Resveratrol, a polyphenol found in grapes, peanut, and cranberry, is an antioxidant [17] with a significant role in reduction of oxidative stress in periodontal structure [18, 19].In addition, this compound has therapeutic effects on the inflammation caused by periodontal pathogens [20].

To date, different surgical and non-surgical treatments have been used for periodontitis; however, few drugs are available for the treatment of periodontitis. Herbal medicines have been used adjunctive to treat inflammatory diseases and recently to treat and prevent periodontitis, too [21, 22]. Since few studies have investigated the clinical effects of resveratrol on improving chronic periodontitis and inflammatory factors, this research aimed to evaluate the effect of resveratrol supplementation as an adjunct to non-surgical treatment of periodontal conditions, including pocket depth (PD), clinical attachment level (CAL), plaque index (PI), bleeding index (BI), and inflammatory factors, such as salivary levels of IL-8 and IL-1β.

## Methods

### Subjects and study design

In this randomized, double-blind clinical trial, 40 periodontitis patients referring to the Periodontics Department of Dental School, Shahid Sadoughi University of Medical Sciences, Yazd, were selected. According to the principles of the Helsinki declaration, after examination and diagnosis of periodontal symptoms and considering inclusion and exclusion criteria, the necessary information was provided, and written consent was obtained. Then, the subjects were randomly assigned to two groups: case (n = 20) and control (n = 20). The patients were free to leave the study at any time they wished.

The inclusion criteria consisted of an age range of 30–60 years and moderate to severe periodontitis. The exclusion criteria consisted of pregnancy, lactation, traveling for > 2 weeks, smoking, taking immunosuppressive medications, taking non-steroidal anti-inflammatory drugs, antihypertensive agents, antibiotics drugs, anti-coagulant drugs like warfarin, taking insulin, and patients treated for uterine, breast, and ovarian cancer, patients with advanced renal and hepatic diseases, those with an allergy to grapes, blackberry, or blueberry, history of periodontitis treatment in the past six months and patients with active oral disease like pemphigus, leukoplakia.

This clinical trial was approved by the Ethics Committee of Shahid Sadoughi University of Medical Sciences under the code IR.SSU.REC.1396.206, and the IRCT Registration code was IRCT20171015036782N6.

### Periodontal examination

Periodontitis patient with stage II (moderate periodontitis) to stage IV (advanced periodontitis with extensive tooth loss) based on clinical attachment loss (CAL) of ≥ 3 mm around the teeth and disease severity according to 2017 international periodontology workshop were selected. As all patients in our study didn’t have full mouth periapical radiography and no risk factor that may affect systemic health according to eligible patients, the grade of periodontitis was estimated with moderate progression rate (grade B).

For all the patients, the probing depth (PD) was measured by the Williams probe, which is a distinctive and calibrated device, at six points of each tooth: mesiobuccal, mid-buccal, distobuccal, mesiolingual, mid-lingual, and distolingual. These areas were coded, and 10 points were randomly selected from at least three quadrants for the post-intervention clinical measurements [19].

Clinical attachment loss (CAL) was also recorded in these areas by measuring the distance between the CEJ (cementoenamel junction) and the pocket depth. In addition, the O’Leary plaque index [23] was recorded after chewing disclosing agent pills, and the gingival bleeding index [24] was calculated 30 s after gentle probing of the sulcus of teeth. All the clinical parameters were performed by the same operator and re-measured four weeks after the intervention to compare with the initial findings.

### Biochemical measurement

Salivary samples of all the patients were collected at baseline and four weeks after the intervention to assess IL-8 and IL-1β levels. All the salivary samples were collected between 9 a.m. and 12 p.m. The patients were asked to refrain from brushing 12 h before sampling and refrain from eating, drinking, and chewing gum two hours before the sampling. To investigate the inflammatory interleukin levels, the unstimulated saliva of the patients was collected before any action. The patients were asked to avoid swallowing their saliva for five minutes, and then the accumulated saliva in the oral cavity floor was passively drooled into sterile disposable test tubes [25]. The samples were then immediately transferred to the laboratory in an ice chamber and centrifuged at 2500 rpm for 20 min at 4 °C. Then the supernatant was frozen at -80ºC until the biochemical tests. The biochemical tests were performed within six months from the moment of freezing. The salivary levels of IL-8 and IL-1β were measured by an ELISA laboratory kit (Karmania PARS Gene, Tehran, Iran).

### Intervention

For all the patients (both case and control groups), scaling and root planning by an ultrasonic scaler (ultrasonic piezo scaler Uds-K, Woodpecker, China) was performed. Patients in the case group received 480 mg (2 capsules) of resveratrol (HERBAFIT Co.) daily for four weeks, while patients in the control group received 480 mg (2 capsules) of placebo containing starch [19]. Patients were asked to take these capsules in the morning after breakfast. During these four weeks, all the patients were contacted three times a week to ensure they took the capsules. All the patients also underwent periodontal examinations and treatment, including scaling and root planning, if needed, during the study. Oral hygiene instructions, including correct tooth brushing and dental flossing, were given to them. After four weeks, the patients were re-examined. All the procedures were done by the same operator.

### Outcome (primary and secondary)

The primary outcome of this study was to determine the CAL and PD before and after the intervention. The secondary outcome of this study was to evaluate the O’Leary plaque index, BI and the mean salivary levels of IL-8 and IL-1β before and after intervention between two groups.

### Sample size calculation

Considering a 5% significance level, 80% test power, and according to the results of a previous study [19] and a standard deviation of S = 0.6, to achieve a significant difference of at least one unit in the mean pocket depth, 20 subjects were included in each group.

### Statistical analysis

SPSS 23.0 was used to compare the results and determine the differences in different experiments. A Kolmogorov-Smirnov test was to assess distribution normality. Also, the chi-squared test, independent-samples t-test, and paired-samples statistics were used to compare the changes between the two groups. Statistical significance was set at P < 0.05.

## Results

In the present study, 40 patients with chronic periodontitis (20 patients in the control group and 20 in the case group) were enrolled after signing consent forms. First, the participants’ mean ages were 44.5 and 41.7 years in the case and control groups, respectively, with no significant difference between the two groups (P = 0.331).

The participants in the case group consisted of 8 males (40%) and 12 females (60%); in the control group, there were 9 males (45%) and 11 females (55%), with no significant difference between the two groups (P = 0.5).

At baseline, the mean distance between the gingival margin and the pocket depth (PD) in the case group was 4.44 mm, with 4.27 mm in the control group, which decreased to 2.82 mm in the case group and 3.2 mm in the control group one month (4 weeks) after the intervention. A comparison of PD values showed a significant decrease in both groups compared to baseline (P = 0.0001), with non-significant changes in the pocket depth in the resveratrol group compared to the control group (P = 0.06) (Tables 1). Pocket closure in case and control group were 95% and 85% respectively.

**Table 1:** Comparison of measured indices between case and control groups before and after intervention

Variable		Case (n = 20) Mean ± SD	Control (n = 20) Mean ± SD	P-value^*^
PD (mm)	B^†^	4.44 ± 0.63	4.27 ± 0.58	0.4
	A^‡^	2.82 ± 0.58	3.2 ± 0.66	0.06
CAL (mm)	B	5.17 ± 0.64	4.97 ± 0.58	0.3
	A	3.37 ± 0.56	3.74 ± 0.6	0.05
BI (%)	B	80.25 ± 15.21	70.80 ± 14.53	0.05
	A	29.10 ± 8.66	32.40 ± 7.29	0.2
PI (%)	B	76.10 ± 13.73	75.60 ± 14.54	0.9
	A	24.05 ± 6.77	34.90 ± 8.68	0.0001
IL-8 (pg/mL)	B	18.67 ± 3.65	16.83 ± 2.63	0.63
	A	17.36 ± 3.03	16.97 ± 1.97	0.07
IL-1β (pg/mL)	B	7.54 ± 3.74	6.24 ± 3.16	0.43
	A	4.34 ± 2.27	4.96 ± 2.67	0.24

^*^P < 0.05 was considered significant at baseline and significant after the intervention using an independent t-test between the two groups.

^†^ Before intervention.

^‡^ After intervention

The mean distance from the CEJ to pocket depth (CAL) at baseline in the case group was 5.17 mm, with 4.97 mm in the control group, which decreased to 3.37 mm in the case group and 3.74 mm in the control group one month (4 weeks) after the intervention. A comparison of the CAL values indicated significant changes in both groups compared to the baseline (P = 0.0001), with no significant decrease after treatment in the case group compared to the control group (P = 0.05) (Tables 1).

The mean of the bleeding index (BI) in the case group was 80.25%, with 70.80% in the control group, which decreased to 29.10% in the case group and 32.40% in the control group one month (4 weeks) after the intervention. A comparison of BI values showed a significant decrease in both groups compared to the baseline (P = 0.0001) and no significant changes after treatment in the resveratrol group compared to the control group (P = 0.2) (Tables 1).

The mean plaque index (PI) in the case group was 76.10%, with 75.60% in the control group, which decreased to 24.05% in the case group and 34.90% in the control group one month (4 weeks) after the intervention. A comparison of PI values showed significant changes in both groups compared to the baseline (P = 0.0001) and a significant decrease after treatment in the resveratrol group compared to the control group (P = 0.00) (Tables 1).

The mean salivary levels of interleukin-8 (IL-8) at baseline in the case and control groups were 18.67 and 16.83 pg/mL in the control group, which increased to 17.36 and 16.97 pg/mL in the case and control groups, respectively, one month (4 weeks) after the intervention. The comparison of IL-8 values showed no significant changes in the case and control groups compared to the baseline (P > 0.05) and no significant changes after treatment with resveratrol (P = 0.07) (Tables 1).

IL-1β salivary levels in the case and control groups were 7.54 and 6.24 pg/mL, respectively, which decreased to 4.34 and 4.96 pg/mL in the case and control group, respectively, one month (4 weeks) after the intervention. A comparison of IL-1β levels showed significant decreases in the case and control groups compared to the baseline (P = 0.0001), with no significant decrease after treatment with resveratrol in the case group (P = 0.24) (Tables 1).

## Discussion

The present research is one of the first studies to investigate the effect of resveratrol supplementation on periodontitis. In this double-blind study with a placebo control, the effectiveness of resveratrol as a supplementary treatment for clinical indicators (PD, CAL, PI, and BI) and salivary inflammatory indicators (IL-8 and IL-1β) was investigated in chronic periodontitis patients without any systemic disease.

According to the results, there were no significant differences between the control and experimental groups in gender and age; in this regard, the two groups were homogenous. According to the comparisons between the two groups, after four weeks of resveratrol supplementation in the experimental group, the only clinical indicator with a significant decrease compared to the control group was plaque index (PI) (P = 0.0001). However, there were no significant differences between the two groups in pocket depth (PD), clinical attachment level (CAL), bleeding index (BI), and IL-8 and IL-1β levels in salivary samples (Table 1). Also, investigating the experimental and control groups independently indicated that the changes in salivary IL-8 levels were not significant in the two groups, with no difference in this indicator before and after the intervention (Table 1). In contrast, the decrease in the remaining indicators, including PI, BI, CAL, PD, and Il-1β after the intervention, was significant compared to the baseline levels in both groups.

In this research, the changes in PD levels were not significantly different between the experimental and control groups, which is different from the results of a study by Zare et al. (2017), who investigated the effect of resveratrol on 43 patients with type 2 diabetes and chronic periodontitis over four weeks [26]. The differences in the findings can be attributed to the systemic diabetes diseases in the samples of Zare et al. and its higher destructive and oxidative effect on the periodontium, which results in a higher effect of resveratrol on this parameter.

Several studies have investigated the effect of non-surgical periodontal treatments, such as oral hygiene instructions, scaling, and root planning, as the key treatments for these patients [27]. The present research also suggested a significant decrease in PD after the intervention in both groups (P = 0.0001). Therefore, non-surgical periodontal treatment played a significant role in both groups.

In the four weeks of the study, CAL decreased by 1.23 mm in the experimental group and 1.8 mm in the control group; these changes are statistically significant (Table 1). Since CAL measures provide a more accurate evaluation of periodontal disease progression, and PD changes cannot provide a reliable prediction of attachment loss [28], it was also evaluated in this study. The results indicated that the decrease in CAL in the resveratrol treatment group compared to the control group was almost significant (P = 0.05) (Table 1). This decrease can result from the secretion of anti-inflammatory and antioxidative factors in the saliva or gingival crevicular fluid (GCF) due to the systemic use of resveratrol and its effect on improving collagen production and attachment to the periodontium and connective tissue. Although the decrease in the average probing depth by 2 mm or higher is usually an indicator of a clinical effect [29], the difference between the two groups (the experimental group: 1.8 mm, and the control group: 1.32 mm) was not clinically significant.

The O’Leary plaque index significantly decreased in both the experimental and control groups compared to the baseline (P = 0.0001), and it also significantly decreased in the experimental group compared to the control group (P = 0.0001) (Table 1). These findings can be justified by the results reported by Millhouse et al., who found that resveratrol had an antimicrobial effect on planktonic bacteria and bacterial biofilms and inhibited the accumulation and activation of neutrophils [30]. Also, Khazaei et al. reported resveratrol’s inhibitory effect on the expression of cell adhesion molecules; it inhibited the endothelial dysfunction caused by *P. gingivalis* lipopolysaccharide and blocked the expression of ICAM-1 and VCAM-1 molecules by inhibiting NF-kappa B [31]. These findings can lead to bacterial plaque rupture around the gingiva. Basu et al. performed a cell study in 2018 and reported that polyphenols could significantly invade the bacteria involved in periodontitis, such as *P. gingivalis*; the viability, replication, and biofilm formation ability of periodontopathogens could be significantly affected by food polyphenols. Daily use of polyphenols can inhibit biofilm formation and decrease bacterial growth speed [12]. The results of a study by Khazaei and Basu can explain the findings of the present study.

In this study, the bleeding index (BI) significantly decreased in both groups after four weeks (P = 0.0001). However, there was no significant difference between the two groups (P = 0.2), which might be due to the significant effect of non-surgical periodontal treatment, frequent oral hygiene instructions, and the Hawthorne effect in both groups that can diminish the effectiveness of resveratrol.

The salivary IL-1β levels decreased significantly in both the experimental and control groups compared to the baseline; however, there was no significant difference between the two groups after the intervention (P˃0.05). Casati et al. (2013) treated 10-week-old rats with 10 mg/kg of daily resveratrol and measured IL-1β, IL-7, and IL-4 levels. They did not observe any significant difference between the two groups in IL-1β and IL-4 levels, but IL-7 levels decreased in the resveratrol treatment group compared to the control group (receiving placebo) [32]. Teles et al. reported no significant difference between the patient and healthy groups in salivary IL-1β level and concentration, and the average salivary IL-1β level could not predict the periodontal condition (healthy or patient) [33]. Their findings are different from the results of the present study because the present study revealed significant changes in salivary IL-1β levels in both groups following the anti-inflammatory treatment and improvements in gingivitis (P = 0.0001).

Several studies have reported an increase in the salivary or gingival crevicular fluid IL-8 levels in inflamed areas compared to healthy areas. In contrast, some other studies have suggested decreased IL-8 levels in inflamed areas. Therefore, the results are contradictory [34]. The present study did not suggest any significant changes in IL-8 levels in the experimental and control groups compared to the baseline and in the comparison between the experimental and control groups (P˃0.05). However, following the intervention, IL-8 levels decreased in the experimental group and increased in the control group. Despite the non-surgical anti-inflammatory treatments in the two groups and resveratrol supplementation in the experimental group, no change was observed in the salivary IL-8 levels, which might be attributed to the shorter period of the study or ineffectiveness of the used dosages of the drug on IL-8 levels.

Although the mentioned evidence suggests the treatment potential of resveratrol in the host inflammatory modulation, the primary mechanism causing this effect is still unknown. Oral treatment with resveratrol has limited bioavailability, and it is quickly affected by metabolism. Its local treatment or injection might have a higher anti-inflammatory effect on the periodontium. Therefore, although the immunologic findings of this study do not suggest the modulation of immune-inflammatory responses by resveratrol, its molecular mechanism should be studied further. The limitations of the present study included the small number of sample size and short follow-up period. Further studies with larger sample size and longer follow up periods needed to explore the impact of resveratrol supplementation on clinical parameters and inflammatory markers in periodontitis patients.

## Conclusion

In view of the above findings and within the limits of present study the results indicated that supplementation with resveratrol in combination with non-surgical periodontal treatment has significant effect on reduction of plaque index (PI) during 4 weeks. Collectively, it seems that using resveratrol along with nonsurgical periodontal treatment may be beneficial in improvement the clinical parameters and inflammatory condition in periodontitis patients.

## Electronic supplementary material

Below is the link to the electronic supplementary material.

Supplementary Material 1

## Data Availability

The data generated and analyzed during the current study are available from the corresponding author on reasonable request.

## Abbreviations

IL-8: Interleukin 8
IL-1β: Interleukin 1 beta
PD: Pocket depth
CAL: Clinical attachment loss
PI: Plaque index (oral hygiene)
BI: Bleeding index

## References

[1] Farzanegan A, Shokuhian M, Jafari S, Shirazi FS, Shahidi M (2019). Anti-histaminic Effects of Resveratrol and Silymarin on Human Gingival fibroblasts. Inflammation.

[2] Bhattarai G, Poudel SB, Kook SH, Lee JC (2016). Resveratrol prevents alveolar bone loss in an experimental rat model of periodontitis. Acta Biomater.

[3] Hajishengallis G, Lamont RJ. (2016). The polymicrobial synergy and dysbiosis model of periodontal disease pathogenesis. In The Human Microbiota and Chronic Disease (eds L.Nibali and B. Henderson).

[4] How KY, Song KP, Chan KG (2016). Porphyromonas gingivalis: an overview of Periodontopathic Pathogen below the Gum line. Front Microbiol.

[5] Dahiya P, Kamal R, Gupta R, Bhardwaj R, Chaudhary K, Kaur S (2013). Reactive oxygen species in periodontitis. J Indian Soc Periodontol.

[6] Chin YT, Hsieh MT, Lin CY, Kuo PJ, Yang YC, Shih YJ (2016). 2,3,5,4’-Tetrahydroxystilbene-2-o-beta-glucoside isolated from Polygoni Multiflori ameliorates the development of Periodontitis. Mediators Inflamm.

[7] Gurgel BC, Duarte PM, Nociti FH, Sallum EA, Casati MZ, Sallum AW (2004). Impact of an anti-inflammatory therapy and its withdrawal on the progression of experimental periodontitis in rats. J Periodontol.

[8] Holzhausen M, Spolidorio DM, Muscara MN, Hebling J, Spolidorio LC (2005). Protective effects of etoricoxib, a selective inhibitor of cyclooxygenase-2, in experimental periodontitis in rats. J Periodontal Res.

[9] Queiroz-Junior CM, Pacheco CM, Maltos KL, Caliari MV, Duarte ID, Francischi JN (2009). Role of systemic and local administration of selective inhibitors of cyclo-oxygenase 1 and 2 in an experimental model of periodontal disease in rats. J Periodontal Res.

[10] Sekino S, Ramberg P, Lindhe J (2005). The effect of systemic administration of ibuprofen in the experimental gingivitis model. J Clin Periodontol.

[11] FitzGerald GA, Patrono C (2001). The coxibs, selective inhibitors of cyclooxygenase-2. N Engl J Med.

[12] Basu A, Masek E, Ebersole JL. Dietary Polyphenols and Periodontitis-A Mini-Review of Literature. Molecules (Basel, Switzerland). 2018;23(7). 10.3390/molecules23071786 PMC6099717 30036945

[13] Castellino G, Mesa F, Cappello F, Benavides-Reyes C, Malfa GA, Cabello I, Magan-Fernandez A (2021). Effects of essential oils and selected Compounds from Lamiaceae Family as Adjutants on the treatment of subjects with Periodontitis and Cardiovascular Risk. Appl Sci.

[14] Bunte K, Hensel A, Beikler T (2019). Polyphenols in the prevention and treatment of periodontal disease: a systematic review of in vivo, ex vivo and in vitro studies. Fitoterapia.

[15] Forouzanfar F, Forouzanfar A, Sathyapalan T, Orafai HM, Sahebkar A (2020). Curcumin for the management of Periodontal Diseases: a review. Curr Pharm Des.

[16] Baima G, Romandini M, Citterio F, Romano F, Aimetti M (2022). Periodontitis and Accelerated Biological aging: a Geroscience Approach. J Dent Res.

[17] Udenigwe CC, Ramprasath VR, Aluko RE, Jones PJ (2008). Potential of resveratrol in anticancer and anti-inflammatory therapy. Nutr Rev.

[18] Bhat KP, Pezzuto JM (2002). Cancer chemopreventive activity of resveratrol. Ann N Y Acad Sci.

[19] Javid AZ, Hormoznejad R, Yousefimanesh HA, Haghighi-Zadeh MH, Zakerkish M (2019). Impact of resveratrol supplementation on inflammatory, antioxidant, and periodontal markers in type 2 diabetic patients with chronic periodontitis. Diabetes Metab Syndr.

[20] Andrade EF, Orlando DR, Araújo AMS, de Andrade J, Azzi DV, de Lima RR et al. Can Resveratrol Treatment Control the Progression of Induced Periodontal Disease? A Systematic Review and Meta-Analysis of Preclinical Studies. Nutrients. 2019;11(5). 10.3390/nu11050953 PMC6566182 31035477

[21] Corrêa MG, Pires PR, Ribeiro FV, Pimentel SZ, Casarin RC, Cirano FR (2017). Systemic treatment with resveratrol and/or curcumin reduces the progression of experimental periodontitis in rats. J Periodontal Res.

[22] Shahidi M, Vaziri F, Haerian A, Farzanegan A, Jafari S, Sharifi R (2017). Proliferative and anti-inflammatory Effects of Resveratrol and silymarin on human gingival fibroblasts: a view to the future. J Dent (Tehran).

[23] O’Leary TJ, Drake RB, Naylor JE. The plaque control record. J Periodontol. 1972 Jan;43(1):38. doi: 10.1902/jop.1972.43.1.38. PMID: 4500182. 10.1902/jop.1972.43.1.38 4500182

[24] Dwarakanath CD. Carranza’s clinical Periodontology-Ebook: third south Asia Edition. Elsevier Health Sciences; 2019.

[25] Navazesh M (1993). Methods for collecting saliva. Ann N Y Acad Sci.

[26] Zare Javid A, Hormoznejad R, Yousefimanesh HA, Zakerkish M, Haghighi-Zadeh MH, Dehghan P (2017). The impact of Resveratrol supplementation on blood glucose, insulin, insulin resistance, triglyceride, and Periodontal markers in type 2 Diabetic patients with chronic Periodontitis. Phytother Res.

[27] Ou L, Li RF (2011). Effect of periodontal treatment on glycosylated hemoglobin levels in elderly patients with periodontal disease and type 2 diabetes. Chin Med J (Engl).

[28] Michalowicz BS, Hodges JS, Pihlstrom BL (2013). Is change in probing depth a reliable predictor of change in clinical attachment loss?. J Am Dent Assoc.

[29] Preshaw PM, Hefti AF, Jepsen S, Etienne D, Walker C, Bradshaw MH (2004). Subantimicrobial dose doxycycline as adjunctive treatment for periodontitis. A review. J Clin Periodontol.

[30] Millhouse E, Jose A, Sherry L, Lappin DF, Patel N, Middleton AM (2014). Development of an in vitro periodontal biofilm model for assessing antimicrobial and host modulatory effects of bioactive molecules. BMC Oral Health.

[31] Khazaei S, Khazaei M, Kazemi S, Yaghini J (2012). Resveratrol as a supplemental treatment for periodontitis. Dent Res J.

[32] Casati MZ, Algayer C, Cardoso da Cruz G, Ribeiro FV, Casarin RC, Pimentel SP (2013). Resveratrol decreases periodontal breakdown and modulates local levels of cytokines during periodontitis in rats. J Periodontol.

[33] Teles R, Likhari V, Socransky S, Haffajee A (2009). Salivary cytokine levels in subjects with chronic periodontitis and in periodontally healthy individuals: a cross-sectional study. J Periodontal Res.

[34] Paul AM, Victor DJ, Gana Prakash PS. Role of Interleukin-8 in Periodontal Disease. International Journal of Clinical Dental Science. 2012;3(2).

