# Supplementary material for: Impact of resveratrol supplementation on clinical parameters and inflammatory markers in patients with chronic periodontitis: a randomized clinical trail
Source: BMC Oral Health. 2023 Mar 27;23:177. doi: 10.1186/s12903-023-02877-4 (PMC10045616; doi:10.1186/s12903-023-02877-4)
Supplement: Supplementary file 1 — Supplementary Material 1 [file 12903_2023_2877_MOESM1_ESM.docx]

**4 weeks follow up**

- Assessing periodontal indices,
- Salvia collecting

**Analysis**

- Laboratory analysis of Salvia by ELISA including levels of 1L-1B, 1L-8
- Analysis of periodontal indices

**Excluded (n=3)**

- 1 discontinue intervention

- 1 adverse effect

- 1 lost to follow

**Excluded (n=3)**

- 1 discontinue intervention

- 2 lost to follow

**Control group (n=23)**

**Intervention group (n=23)**

**Baseline visit**

Examining and assessing Periodontal indices, Saliva collecting, giving resveratrol capsules

**Required subjects (n=48):**

[Taking consent form, subject information form

Periodontal examination]

**Excluded (n=2)**

**Allocation (n=46)**

**Baseline visit**

Examining and assessing Periodontal indices, Saliva collecting, giving resveratrol capsules
